# Dissipation, Residue and Human Dietary Risk Assessment of Pyraclostrobin and Cyazofamid in Grapes Using an HPLC-UV Detector

**DOI:** 10.3390/foods13020314

**Published:** 2024-01-18

**Authors:** Peiying Zhao, Rong Liu, Longfei Yuan

**Affiliations:** 1 State Key Laboratory of Integrated Management of Pest Insects and Rodents, Institute of Zoology, Chinese Academy of Sciences, Beijing 100101, China; zhaopy2020@163.com; 2 Institute of Crop Sciences, Chinese Academy of Agricultural Sciences, Beijing 100081, China

**Keywords:** pyraclostrobin, cyazofamid, CCIM, dissipation behavior, residue, dietary risk assessment, grape

## Abstract

Pyraclostrobin is a new broad-spectrum methoxyacrylic acid fungicide. Cyazofamid is a new selective foliar spray acaricide. Here, we studied the degradation rate and final residues of pyraclostrobin and cyazofamid in grape and evaluated their dietary risk to consumers. The average recoveries of pyraclostrobin ether ester, cyazofamid and cyazofamid metabolite (CCIM) in grapes were 84–94%, 92–98% and 99–104%, respectively. The relative standard deviations (RSDs) were 6.0–20.3%, 2.4–10.5% and 1.3–4.0%, respectively, and the LOQs were all 0.05 mg/kg. The digestion dynamics of the experimental sites were in accordance with the first-order kinetic equation. The degradation half-lives of pyraclostrobin ether ester and cyazofamid were 17.8 d–28.9 d and 4.3 d–7.8 d, respectively. The final residues of pyraclostrobin ether ester, cyazofamid and CCIM in grapes were <0.05–1.88 mg/kg, <0.05–0.31 mg/kg and <0.05–0.47 mg/kg, respectively. Using probability models, the total chronic risk values for pyraclostrobin and cyazofamid were calculated to be 0.112–189.617% and 0.021–1.714%, respectively. The results of the contribution analysis indicate that pyraclostrobin poses a much greater risk to Chinese consumers than cyazofamid, especially to children and adolescents, who have a significantly greater risk than adults. This suggests that more consideration should be given to the cumulative risk of compounds for vulnerable groups in the future.

## 1. Introduction

China has a history of over 2000 years of grape cultivation [1]. It is estimated that the surface area of the world’s vineyards in 2022 was 7.3 mha, while the area of Chinese vineyards was 785 kha, representing the third greatest area of vineyards in the world [2]. Frost mold is a major threat to grape cultivation, leading to severe yield losses [3]. Therefore, fungicides have been universally used in agricultural production to reduce plant diseases [4]. Pyraclostrobin, cyazofamid and cyazofamid metabolite (CCIM) are currently some of the most important fungicides [5,6,7] and are mainly used for the treatment of fungal diseases [4,8], especially for the prevention and treatment of downy mildew with significant effects [6,9]. For large-scale applications, the dietary exposure risks of pyraclostrobin, cyazofamid and cyazofamid metabolite (CCIM) to consumers have also attracted attention [10]. Pyraclostrobin, methyl [2-[1-(4-chlorophenyl)-1h-pyrazol-3-yloxymethyl]phenyl]methoxycarbamate, has broad-spectrum properties [6] and is a methoxyacrylate fungicide able to effectively control fungal diseases [11,12]. By blocking the electron transfer between cytochrome b and C1 [11], it can inhibit mitochondrial respiration, preventing mitochondria from producing and supplying the energy required for normal cell metabolism and ultimately leading to cell death [13]. The chemical names of cyazofamid and its metabolite (CCIM) are 4-chloro-2-cyano-N,N-dimethyl-5-(4-methylphenyl)-1H-imidazole-1-sulfonamide and 4-chloro-5-p-tolylimidazole-2-carbonitrile, respectively. Cyazofamid and CCIM are sulfonamide-based fungicides with a new and unique way of action that inhibits the Qi site (ubiquinone reduction site) of cytochrome bc1 enzyme complex III in the mitochondrial respiratory chain [9,14,15,16], affecting all growth stages of oomycetes [10,17]. The molecular structural formulas of the above three compounds are depicted in Figure S1.

Pyraclostrobin exhibits a certain level of toxicity towards zebrafish, daphnia magna, earthworms, brachydanio rerio, xenopus laevis and rana nigromaculata [18,19,20,21]. The 96 h median lethal concentration of pyraclostrobin against zebrafish has been determined to be 61 μg/L [22], while the median effective concentration (EC50) for its toxicity towards lampsilis siliquoidea has been found to be less than 50 mg/L [8]. Furthermore, pyraclostrobin has been associated with adverse effects on reproductive and developmental abilities [19,23]. It also causes damage to drosophila DNA [24]. Meanwhile, studies have shown that pyraclostrobin can have harmful effects on bee adipocytes and pericardial cells, damaging their detoxification and immune defense [25,26]. Zhang also proposed that pyraclostrobin can cause liver DNA damage and has toxic effects on antioxidant enzyme activity in zebrafish [18]. Yoshizawa proposed that pyraclostrobin can also have an impact on liver vitality in humans, rodents, rabbits, dogs and other animals [27]. At the same time, pyraclostrobin can also affect human HepG2 cells [28] and have adverse effects on mitochondrial function in human liver cells [29], posing a potential risk of hepatotoxicity. At the same time, there is a potential risk of genetic toxicity to human peripheral blood mononuclear cells and peripheral blood lymphocytes due to the presence of pyraclostrobin [30,31]. In contrast, cyazofamid exhibits low toxicity towards both humans and the environment, thereby reducing the likelihood of causing pollution [32]. However, it is susceptible to residue accumulation, potentially leading to soil and water contamination through its long-term usage. Additionally, male rats have high renal toxicity when exposed to cyazofamid. Moreover, CCIM has been demonstrated to possess a higher level of acute toxicity compared to that of cyazofamid [33].

As is widely acknowledged, the extensive and prolonged utilization of pesticides has resulted in food and feed contamination. Therefore, on-site dissipation and final residue analyses of diverse pesticides are imperative for ensuring food safety and safeguarding the environment. Numerous studies have previously reported residual analysis methods for pyraclostrobin, cyazofamid and CCIM in various crops. Pang proposed a liquid chromatography/mass spectrometry method to determine pyraclostrobin, cyazofamid and CCIM in grape samples [10]. Paper spray ionization mass spectrometry (PSI-MS) has also been employed to rapidly quantify pyraclostrobin residues in tomatoes [12]; UHPLC-MS/MS was used to detect residues in chili [34] and cucumber [8]. Lee developed a rapid and robust LC-MS/MS method for analyzing environmental samples (soil and water) as well as multiple crops (apples, citrus fruits, kimchi, green peppers, potatoes and soybeans) [35]. The LC-MS/MS method can also be utilized to determine the residues of cyazofamid and CCIM in grapes [36], Korean cabbage [37], tomatoes [14] and soil.

With the increasing use of pesticides, there are concerns about whether their excessive use will cause harm to the human body. Therefore, it is necessary to conduct dietary risk assessments of pesticide residues in fruits, which can determine whether they will cause potential health problems and provide a scientific basis for safe production and maximum residue limits [38,39]. The dietary risks of pyraclostrobin and cyazofamid have been researched. In 2021, Zhao determined the residual levels of pyraclostrobin in wheat and evaluated calculations of risk quotas [40]. In 2023, Li conducted a study on the risk quotas of cyazofamid in turnip, onion and romaine lettuce [41]. However, these articles did not focus on dietary risks through probability models.

This study aimed to develop an accurate, straightforward and sensitive method using QuEChERS extraction and high-pressure liquid chromatography (HPLC) coupled with a UV detector for the determination of pyraclostrobin, cyazofamid and CCIM residues in grapes from ten representative regions in China. In addition, attempts were made to calculate the acute and chronic dietary intake risks of pyraclostrobin and cyazofamid using deterministic and probabilistic models based on the final residual levels and toxicological data. The findings of this study can provide valuable guidance for the rational application of pyraclostrobin and cyazofamid in grape production.

## 2. Materials and Methods

### 2.1. Reagents and Standards

Pyraclostrobin standard with a purity of 99% and cyazofamid standard with a purity of 99.5% were purchased from Dr. Ehrenstorfer GmbH (Augsburg, Germany). CCIM standard with a purity of 95% was purchased from Beijing Sunshine Furunde Technology Trading Co., Ltd. (Beijing, China). Chromatography-grade pure acetonitrile and chromatography-grade pure methanol was purchased from Fisher Chemical Co., Ltd. (Waltham, MA, USA). Analytical-grade sodium chloride was purchased from Beijing Tongguang Fine Chemicals Company (Beijing, China). Chromatographic-grade formic acid was provided by CNW Technology (Shanghai, China). The purification tube containing primary secondary amine (PSA) filler was provided by Agela Technologies (Tianjin, China). The 35% pyraclostrobin/cyazofamid suspension concentrate (SC) was purchased from Chengdu Kelilong Biochemical Co., Ltd. (Chengdu, China).

We accurately weighed about 10 mg of pyrazolamide standard, cyazofamid standard and CCIM standard; dissolved them in 10 mL of acetonitrile to obtain 1000 mg/L standard solution; and store the prepared standard solution in a refrigerator at 4 °C in the dark. We accurately weighed 2.5 mL of 1000 mg/L standard solution and mixed it with acetonitrile. Appropriate amount of standard mixed solution was accurately transferred and diluted with blank grape matrix extract to prepare matrix-matched standard solutions of 0.05, 0.10, 0.20, 0.50, 1.0, 2.0 and 5.0 mg/L for sample quantification.

### 2.2. Extraction and Purification Process of the Samples

A total of 10 g (±0.05 g) of homogenized grape sample was meticulously weighed and placed into a 50 mL centrifuge tube. A total of 10 mL of chromatographic-grade acetonitrile was added to this tube, followed by 15 min sonication in a water bath. To this mixture, 6 g of NaCl was added, thoroughly shaken and then centrifuged at 3000 rpm for 5 min. From the resulting solution, 1.5 mL of the upper clear liquid was extracted and transferred to a 2 mL centrifuge purification tube containing 50 mg of primary secondary amine (PSA). It was vortexed at 2500 rpm for 2 min. After centrifugation at 10,000 rpm for an additional 2 min, the supernatant was filtered through a 0.22 μm nylon filter membrane for subsequent analysis using an HPLC-UV detector. This experiment uses acetonitrile extraction, PSA tube purification and HPLC detection, which belong to the QuEChERS method. Therefore, this experiment can more conveniently and quickly perform sample detection.

### 2.3. Instrumentation

Various laboratory instruments were used in the pretreatment of samples and extraction of analytes, including a desktop centrifuge (model SC-3612) from Anhui Zhongke Zhongjia Instrument Co., Ltd. (Anhui, China), a high-speed centrifuge (model Pico 17) from Thermo Scientific (City of San Jose, CA, USA), an ultrasonic cleaner (model KQ-600) from Kunshan UltrasoundInstrument Co., Ltd. (Jiangsu, China), a laboratory water purification system (S30UV type) from Shanghai Hetai Instrument Co., Ltd. (Shanghai, China), and an eddy current mixer (model MTV-100) from Hangzhou Aosheng (Zhejiang, China). In the detection process, the analyte was monitored using the HPLC 1200 UV detector manufactured by Agilent Technologies (USA) Co., Ltd. (City of Santa Clara, CA, USA).

Pyraclostrobin, cyazofamid and CCIM were attained using high-performance liquid chromatography (Agilent 1200, City of Santa Clara, CA, USA) with a variable UV detector set to 280 nm. Analytical column was Agilent 5 HC-C18 m (250 mm × 4.6 mm i.d. and 5 μm particle size). The mobile phase consisted of 0.1% formic acid water/ acetonitrile (35:65, *v*/*v*). The column temperature was maintained at 35 °C and the flow rate was 1 mL/min with a run time of 20 min, as shown in Table S1.

### 2.4. Field Trial Tests

According to the Guidelines for Pesticide Residue Testing (NY/T 788-2018) [42] issued by the Ministry of Agriculture and Rural Affairs of China, field experiments were designed. Field experiments were conducted in 10 diverse locations in China, including Daxing, Beijing (116.13 E, 39.26 N; warm temperate semi-humid continental monsoon climate), Suzhou, Anhui (116.09 E, 33.18 N; warm temperate semi-humid monsoon climate zone), Nanning, Guangxi (107.20 E, 22.47 N; subtropical monsoon climate), Xinxiang, Henan (113.55 E, 35.20 N; continental warm temperate monsoon climate), Duyun, Guizhou (107.31 E, 26.15 N; subtropical humid monsoon climate), Changsha, Hunan (112.59 E, 28.12 N; subtropical humid monsoon climate), Haerbing, Heilongjiang (125.42 E, 44.04 N; northern temperate monsoon climate), Zibo, Shandong (117.32 E, 35.55 N; temperate monsoon climate), Jinzhong, Shanxi (112.71 E, 37.69 N; temperate continental monsoon climate), and Hangzhou, Zhejiang (118.21 E, 29.11 N; subtropical monsoon climate). At least 10 (at least 1 kg) normally growing and healthy grapes were harvested from more than 8 grapevines, and each test was conducted in triplicate. The spraying and sample processing methods for the blank control experimental site were the same as those for the experimental site, and protective zones were established between different plots to prevent contamination between the experimental samples. We applied pesticides using the water spray method. We applied pesticides once when the disease was induced. The dosage of pesticide formulation was 4000 times liquid (1:4000) (87.5 mg a.i./kg), and the final residual samples were collected 7, 14 and 21 days after the last application. Dynamic digestion experiments were conducted in Guangxi and Zhejiang provinces, with grape samples collected 0, 3, 7, 14, 21 and 28 days after the last application of pesticides. All of the residual samples to be tested were frozen and stored at −20 °C until detection was complete.

### 2.5. Deterministic Model

Matrix effect (ME) is evaluated by the ratio of the slope of the matrix standard curve to the slope of the solvent standard curve, as shown in Equation (1) [43]:

(1) $$\left|\mathrm{ME}\right|\%=\left(K_{1}/K_{2}-1\right)\times 100\%$$

Among these, $K_{1}$ represents the slope of the grape matrix standard curve, and $K_{2}$ represents the slope of the solvent standard curve $\left|\mathrm{ME}\right|\%\geq 20\%$ indicates a significant matrix effect. $\left|\mathrm{ME}\right|\%\leq 20\%$ indicates that the matrix effect is not significant.

The first-order model and double exponential model fit the dissipation patterns of pyraclostrobin, cyazofamid and CCIM in crops, and the degradation rate (DT%) was always related to the initial concentration, as shown in Equations (2)–(4) [44]:

(2) $$C_{t}=C_{0}{\;e}^{-\mathrm{kt}}$$

(3) $$\mathrm{DT}\%=\left(C_{0}-C_{t}\right)/C_{0}$$

(4) $$T_{1/2}=\ln2/K=0.693/K$$

$C_{0}$ (mg/kg) is the initial concentration, $C_{t}$ (mg/kg) is the pesticide residue concentration at time t (day), K is the degradation rate constant (day^−1^) and $T_{1/2}$ (day) is the pesticide degradation half-life determined based on the k value.

Deterministic models can be used to evaluate acute and chronic dietary risks, with the merit of being convenient and effective.

The acceptability of acute dietary intake risk was estimated using National/International Estimated Short-term Intakes (NESTI/IESTI, Equation (5)) and the percentage of acute reference dose (%ARfD, Equation (6)), respectively. The calculation formulas of NESTI and %ARfD were as follows [44]:

(5) $$\mathrm{NESTI}=\frac{\mathrm{LP}\times \mathrm{HR}\times v}{\mathrm{bw}}$$

(6) $$\%\mathrm{ARfD}=\frac{\mathrm{NESTI}}{\mathrm{ARfD}}\times 100\%$$

where HR (mg/kg) is the highest residual value of the edible part of grapes measured in the experiment. v is the coefficient of variation of grapes, which is 3 [44]. The full name of LP (g/d) is the large portion, which refers to the daily food consumption that can cover 97.5% of consumers. bw (kg), also known as bodyweight, refers the average body weight of the population subgroups [44].

Risk quotient (RQ) is an important indicator used to evaluate whether pesticide residues will affect consumers’ dietary risks. RQ is calculated by dividing the National/International Estimated Daily Intake (NEDI/IEDI) by the acceptable daily intake (ADI). When NEDI > ADI, it means that this pesticide may pose certain risks in consumers’ daily diets [45]. The calculation formulas are as follows [44]:

(7) $$\mathrm{EDI}=\sum {(\mathrm{STMR}}_{i}{(\mathrm{STMR}}_{i}{-P)\times F}_{i})/\mathrm{bw}$$

(8) $$\mathrm{RQ}=\mathrm{NEDI}/\left(\mathrm{ADI}\times \mathrm{bw}\right)\times 100\%$$

(9) $$\%\mathrm{ADI}=\left(\mathrm{EDI}/\mathrm{ADI}\right)\times 100\%$$

Among them, ${\mathrm{STMR}}_{i}$ (mg/kg) refers to the median monitoring of pesticide residues in registered food in China. P is the total number of investigated foods. $F_{i}$ (Kg/day) is the average daily intake of grape in China. The ADI value of pyraclostrobin is 0.03 mg/kg bw, and the ADI value of cyazofamid is 0.2 mg/kg bw, sourced from National Food Safety Standard for Maximum Residue Limits for Pesticides in Food (GB 2763–2021) [46]. It the current mandatory national standard for the maximum residue limit of pesticides in food in China. At the same time, the above ADI values are those accepted in the JMPR (Joint FAO/WHO Meeting on Pesticide Residues) [47,48]. The main objective of JMPR is to conduct pesticide residue assessments and propose globally consistent recommendations related to pesticide residues.

### 2.6. Cumulative Dietary Risk Assessment on Probabilistic Method

Probability models estimate dietary intake by calculating probability theory and statistical data [49]. Even though this calculation requires a large amount of supporting data and has certain application constraints, probability models can analyze the uncertainty of exposure levels by fitting residual data and consumption data distributions. They have gradually become a hot research topic in the field of food safety risk assessments in recent years [50]. Their formula is as follows [49]:

(10) $$y_{i}=\sum_{k=1}^{k}\left(x_{\mathrm{ik}}\times c_{\mathrm{ik}}\right)/{\mathrm{bw}}_{i}$$

where $y_{i}$ represents the pesticide intake of the i-th group. k refers to the total number of surveyed foods. $x_{\mathrm{ik}}$ is the food consumption of group I and $c_{\mathrm{ik}}$ refers to the amount of pesticide residues in this type of food. ${\mathrm{bw}}_{i}$ represents to the body weight of different age groups. These data are from the Report on the Nutrition and Health Status of Chinese Residents (2002). Dietary risk is evaluated by the ratio of $y_{i}$ to ADI and ARfD. $x_{\mathrm{ik}}$ and $c_{\mathrm{ik}}$ were fitted to a lognormal distribution, and Monte Carlo random sampling was performed using @RISK 7.5 software (Palisade, Ithaca, NY, USA) to estimate the probability distribution of $y_{i}$ under different uncertainties. A thousand Monte Carlo simulation iterations in the @RISK 7.5 software were completed (Palisade, Ithaca, NY, USA). We divided consumers into different research groups based on their age, gender, and region to calculate the probability of acute risk. The results of probabilistic models are more accurate and in line with the real situation and can provide uncertainty in terms of risk outcomes [51,52]. The accuracy of probabilistic models is reflected in the estimation process of dietary exposure, which no longer uses single statistical point values such as STMR or HR for simulations. Instead, it is based on probability distribution (such as lognormal distribution) or Monte Carlo resampling methods to more accurately assign values to consumption data, pesticide residue data and even individual consumer data in order to improve the accuracy of risk fitting results and obtain uncertainty of the results [49,51,53].

## 3. Results and Discussion

### 3.1. Instrument Results

**Figure 1:**
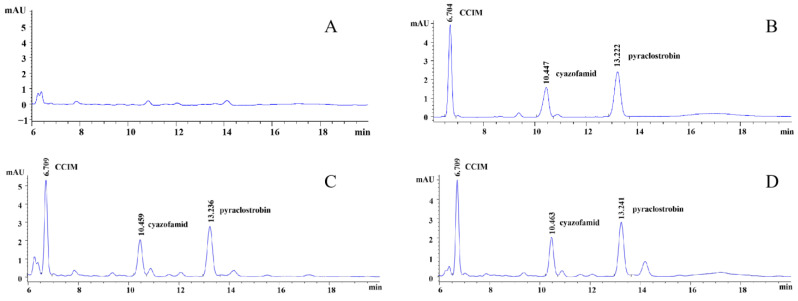
Liquid chromatograms of pyraclostrobin, cyazofamid and CCIM. (**A**) grape blank sample; (**B**) pyraclostrobin, cyazofamid and CCIM solvent standard of 0.05 mg/L; (**C**) pyraclostrobin, cyazofamid and CCIM matrix standard of 0.05 mg/L; (**D**) addition and recovery of 0.05 mg/kg of pyraclostrobin, cyazofamide and CCIM in grapes.

The retention time of pyraclostrobin is 13.236 min, the retention time of cyazofamid is 10.459 min and the retention time of CCIM is 6.709 min, all of which have no interference with one another. The chromatograms of pyraclostrobin, cyazofamid and CCIM are shown in Figure 1.

### 3.2. Method Validation

The method of this experiment is judged by the recovery rate and the RSD value is measured by the solvent and matrix addition recovery, as well as the influence of the matrix. As shown in Table S2, the spiked levels of 0.05, 0.50 and 2.0 mg/kg, the average recovery rate of pyraclostrobin in the grape solvent standard were 84–94%, with a relative standard deviation (RSD) of 6.0–20.3%. At the spiked levels of 0.05, 0.50 and 1.0 mg/kg, the average recovery rates of cyazofamid in the grape solvent standard were 92–98%, with an RSD of 2.4–10.5%. The average recovery rate of the CCIM in the grape solvent standard is 99–104%, with an RSD of 1.3–4.0%. The average recovery rate of the pyraclostrobin in the grape matrix standard was 98–99%, with a relative standard deviation (RSD) of 2.4–19.8%. At the spiked levels of 0.05, 0.50 and 1.0 mg/kg, the average recovery rates of the cyazofamid in the grape matrix standard were 84–100%, with an RSD of 1.9–8.1%. The average recovery rate of the CCIM in the grape matrix standard was 96–102%, with an RSD of 0.5–3.3%. The above results indicate that this method is accurate and precise in both solvent standard and matrix standard detection.

According to the recycling experiment, under the above experimental conditions, the minimum detection levels of pyraclostrobin, cyazofamid and CCIM were 1.0 ng. The minimum content of pyraclostrobin, cyazofamid and CCIM that can be detected by this method in grapes is 0.05 mg/kg. Therefore, according to the above definition, the quantitative limit of this method is to add and recover a minimum concentration of 0.05 mg/kg.

The regression equations of the standard curve of pyraclostrobin in the solvent and grape matrices were y = 75.023x − 0.1507, r = 1 and y = 79.557x + 0.8196, r = 0.9999, respectively. The regression equations of the standard curve of cyazofamid in the solvent and grape matrices were y = 46.077x − 0.5829, r = 0.9999 and y = 44.765x + 0.4464, r = 1, respectively. The regression equations of the standard curve of CCIM in the solvent and grape matrices were y = 83.679x − 1.0367, r = 0.9998 and y = 79.642x + 0.9445, r = 0.9999, respectively. According to formula (1), the matrix effect (ME) of the pyraclostrobin, cyazofamid and CCIM pesticides were calculated to be 6%, 3% and 5%, respectively. From this, it can be seen that these three pesticides have no significant impact on the substrate of grapes. Therefore, this study adopts the solvent standard external standard method for qualitative and quantitative analyses, which is more convenient and cost effective.

### 3.3. Dissipation Behavior

**Figure 2:**
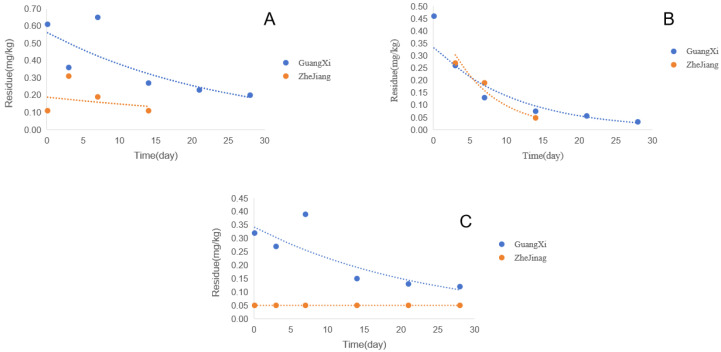
Degradation rates of three compounds in grape samples in Guangxi and Zhejiang: (**A**) pyraclostrobin, (**B**) cyazofamid and (**C**) CCIM.

The degradation rate of pesticides can reflect their dissipation rate, which has a significant impact on the study of consumer exposure to these pesticides over a long period of time. Most of the pesticide content decreases over time after its application, and these two fungicides also conform to this characteristic (Figure 2), with their dissipation processes following first-order reaction kinetics:

The initial deposition of pyraclostrobin was in the range of <0.05–0.65 mg/kg, with a residual dissipation rate of 65–69% after 28 days and a half-life ranging from 17.8 d to 28.9 d. This conclusion is consistent with the half-life of pyraclostrobin in grapes measured by Chen, which was 17.8–25.9 days [54], and Pang’s conclusion that pyraclostrobin in grapes has a half-life of 21 days [10]. The initial deposition of cyazofamid was in the range of <0.05–0.46 mg/kg, with a residual dissipation rate of 71–88% after 28 days and a half-life ranging from 4.3 d to 7.8 d. This detection result is also consistent with Li’s proposed half-life range of 5.3–8.7 days for cyazofamid in turnip, onion and romaine lettuce [41]. The initial deposition of CCIM is in the range of <0.05–0.32 mg/kg, with a residue of 69% after 28 days and a half-life of 16.9 d. The soil types in the experimental areas of Nanning City, Guangxi and Hangzhou City, Zhejiang Province are loam soil and tidal soil, with pH values of 6.3 and 5.33, organic matter contents of 1.7% and 1.17%, and annual average temperatures of 21.6 °C and 17.8 °C, respectively. Therefore, the different degradation rates and initial deposition of pesticides may be attributed to various factors, such as the soil type, pH value and organic matter content at different experimental sites, as well as the temperature and precipitation at the experimental sites. There are also studies indicating that under the same environment, different physicochemical properties, such as the bio-availability and efficacy of pesticides, may also lead to different pesticide degradation rates [55]. From the above experimental data, it can be concluded that the half-life of pyraclostrobin is relatively long. Studies have shown that compounds with long half-lives may lead to cumulative exposure levels higher than initially estimated [56]. They may also cause consumers to have relatively high levels of pesticide residues when purchasing crops. Therefore, the method of applying pyraclostrobin in the early stage of an epidemic could be adopted to increase the number of days between the last application and the time of harvesting and reduce the consumption of pesticide residues by consumers. In addition, studies have shown that pyraclostrobin can be alternately applied with pyrimethanil, procymidone and cyprodinil, which can reduce the half-life of pesticides [57].

### 3.4. Final Residue Testing

According to the effective method of pesticide application in Section 2.4 above, the harvest intervals were set at 7, 14 and 21 d, respectively. The final residues of pyraclostrobin, cyazofamid and CCIM in grape were determined by the standard residue method. At the time of harvesting, the residue levels of pyraclostrobin in grape were <0.05–1.88 mg/kg for the first interval, <0.05–1.09 mg/kg for the second interval and <0.05–0.73 mg/kg for the third interval. For cyazofamid in grape, they were <0.05–0.31 mg/kg, <0.05–0.15 mg/kg and <0.05–0.24 mg/kg, respectively. The total residues of the cyazofamid (the cyazofamid plus its metabolite cyazofamid-diazee, as calculated by the cyazofamid) in grape at the time of harvesting were <0.1–0.78 mg/kg, <0.1–0.29 mg/kg and <0.1–0.28 mg/kg. Therefore, at the pre-harvest interval (PHI) of seven days, the final residues of pyraclostrobin and cyazofamid in grape were lower than the MRL set by China (2.0 mg/kg and 1.0 mg/kg, respectively) [46]. These findings provide valuable insights for proper usage guidelines for pyraclostrobin/cyazofamid SC on grapes.

### 3.5. Acute Dietary Risk Assessment

The ARfD of pyraclostrobin in the JMPR report is 0.05 mg/kg bw [48]. According to the JMPR report, the committee considers that the ARfD value for establishing cyazofamid based on its metabolite CCIM is 0.2 mg/kg bw [47]. The LP of children is 0.366 kg/person, the LP of women of childbearing age is 0.297 kg/person and the LP of the general population is 0.570 kg/person. The weight of children is 16.14 kg, the weight of women of childbearing age is 52.6 kg and the weight of the general population is 53.23 kg [58]. Through residual experiments, the HR of pyraclostrobin in grapes was found to be 1.88 mg/kg, and the HR of cyazofamid in grapes was found to be 0.78 mg/kg. By integrating the HR values of pyraclostrobin and cyazofamid obtained from the final residue test with the LP and U values representing grape consumption in specific subgroups of the Chinese population [59], we calculated the NESTI/IESTI and compared it with the ARfD to assess the acute risk of the model analysis. The dietary risk assessment data are presented in Table S3.

**Figure 3:**
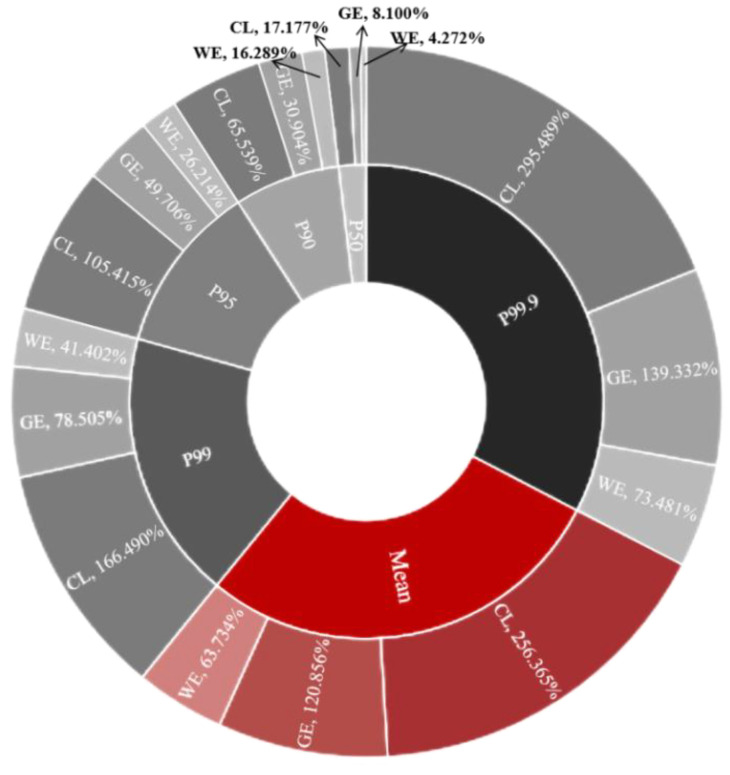
Acute dietary risk assessment results of pyraclostrobin deterministic model and probabilistic model: CL indicates children, GE indicates general people and WE indicates women of childbearing age.

**Figure 4:**
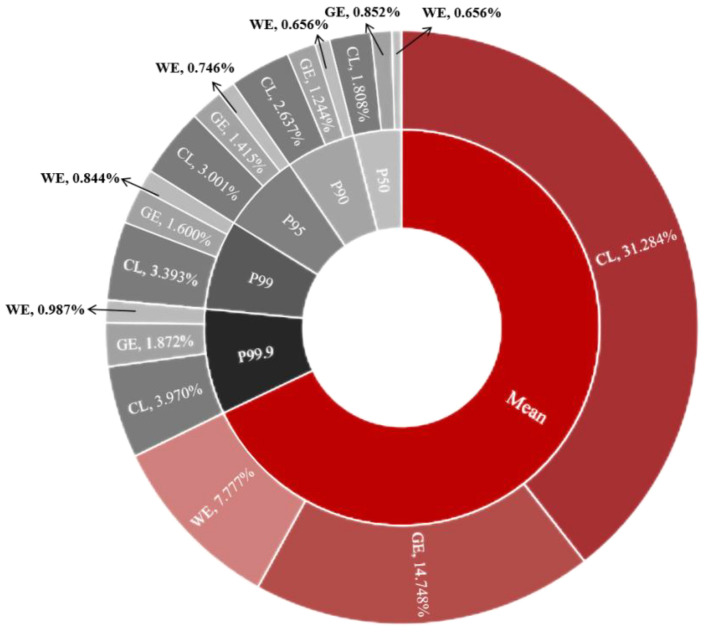
Acute dietary risk assessment results of cyazofamid deterministic model and probabilistic model: CL indicates children, GE indicates general people and WE indicates women of childbearing age.

As depicted in Figure 3 and Figure 4, we evaluated the acute dietary exposure risks of pyraclostrobin and cyazofamid in grapes for three population groups: the general population, children and women of childbearing age. The analysis shows that the %ARfD values for these groups are as follows: the general population—120.86%, the children’s group—256.36% and the women of childbearing age—63.73%. These findings indicate that the ingestion of pyraclostrobin through dietary channels poses unacceptable long-term risks to consumers, especially children and women of childbearing age. For cyazofamid in grapes among different population groups (the general population, children and women of childbearing age), the %ARfD values were found to be 12.54%, 26.59% and 6.61%, respectively, which were all lower than 100%. This suggests that the acute dietary risk associated with cyazofamid intake from grapes is acceptable for Chinese residents. The results of the probability model demonstrate that at all levels considered, pediatric exposure presents a higher risk for children aged one to six than that for women of childbearing age between 14 and 50 years or the general population as a whole. Specifically, at the P95 level, pediatric pyraclostrobin exposure exceeds 100%. By the P99.9 level, children’s dietary risk reaches an alarming rate of 295.489%. The probability model of cyazofamid calculates a relatively low risk value, with an acute binary risk of 0.450–3.970% at each level. None of these exceed 100%, indicating that cyazofamid will not cause dietary harm to consumers. However, it can also be clearly observed that the risk value for children is higher than that for the general population and women of childbearing age. The higher intake of grapes per unit weight observed among children compared to the general population contributed significantly to this disparity. Acute residual exposure levels generally only consider the maximum amount of pesticide residue in one agricultural product (food) [52], which may be significantly higher than the average level [45]. Compared to typical long-term or average consumption, the amount of food consumed at one time may be very large, and the residual amount of food may be much higher than the average level [45,60]. The acute toxicity of pesticides in one’s diet may cause blood toxicity, immunotoxicity, neurotoxicity and hepatorenal toxicity, as well as have endocrine and developmental effects on consumers [61]. According to PPDB queries, it can be inferred that pyraclostrobin is harmful to human reproductive and developmental abilities [23], which may have adverse effects on vulnerable groups, especially children. In the Introduction section, it is also mentioned that pyraclostrobin may have adverse effects, such as hepatotoxicity, on human HepG2 cells [28,29]. Therefore, particular attention should be paid to the short-term risk of acute dietary exposure in children in order to mitigate the health effects resulting from the expansion of joint exposure risks. People should clean up before eating grapes to reduce the risk of children coming into contact with pesticides in their diet. Simultaneously, emphasis should also be placed on assessing the acute dietary risk of pesticide exposure across different crops.

### 3.6. Chronic Dietary Risk Assessment

Standard testing was conducted to determine the median pesticide residue levels of three compounds in the grape matrices. Using deterministic and probabilistic models, food consumption data, the weights of different foods and supervised trials showing the median residue (STMR) values for different populations, we systematically compared the chronic dietary risk across different regions, ages and genders. After 7 days, the median residue level of pyraclostrobin was found to be 0.13 mg/kg, while the sum of cyazofamid and its metabolite CCIM (calculated as cyazofamid) was determined to be 0.11 mg/kg. The STMR of pyraclostrobin in grapes was found to be 0.13 mg/kg, and the STMR of cyazofamid in grapes was found to be 0.11 mg/kg.

**Figure 5:**
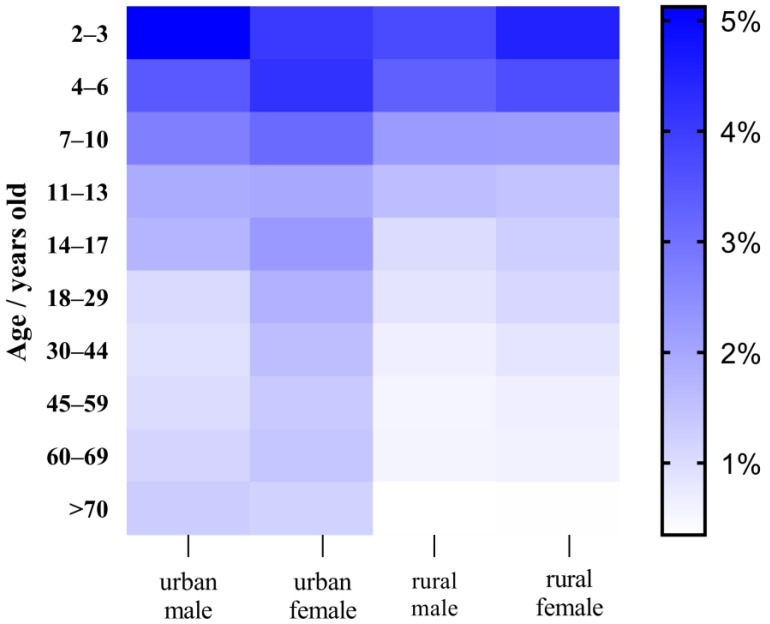
Heat map of chronic dietary risks of pyraclostrobin.

**Figure 6:**
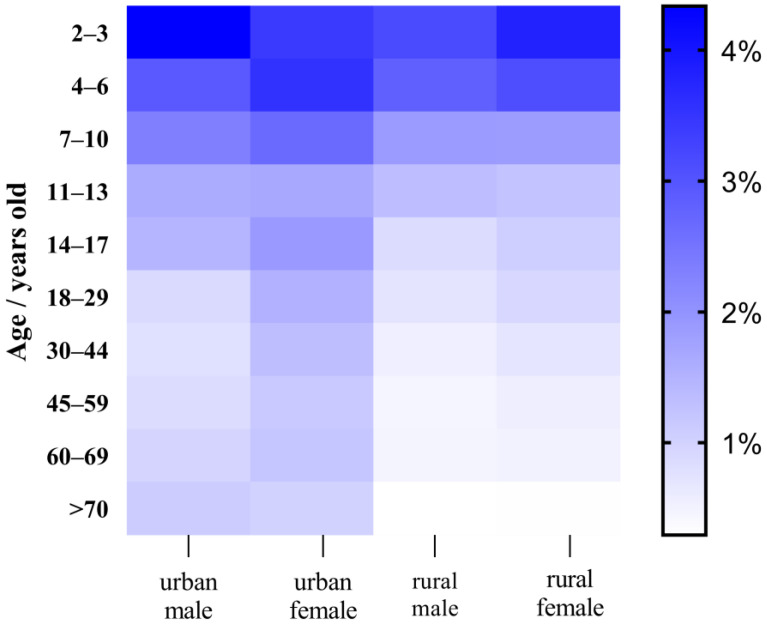
Heat map of chronic dietary risks of cyazofamid.

As shown in Figure 5 and Figure 6, urban men had a chronic dietary risk ranging from 0.926 to 5.122% for pyraclostrobin, urban women’s risk ranged from 1.176 to 4.027%, rural men’s risk ranged from 0.348 to 3.729% and rural women’s risk ranged from 0.352 to 4.484%. The chronic dietary risk associated with cyazofamid and its metabolites for urban men varied between 0.784 and 4.334%, for urban women between 0.995 and 3.529%, for rural men between 0.348 and 3.729%, and for rural women between 0.352 and 4.484%. The deterministic model of the chronic risk of pyraclostrobin in grapes is 0.348–5.122%, and the chronic deterministic risk of cyazofamid is 0.249–4.334%. These values are close to the data calculated by the P90 in the probability model of the chronic dietary risk. As shown in Tables S4 and S5, in probability models, the total chronic risk values for pyraclostrobin and cyazofamid were calculated to be 0.112–189.617% and 0.021–1.714%, respectively. There are still cases in which the risk value of pyraclostrobin exceeds 100%, indicating the possibility of endangering consumer health. Women were at a higher risk than men, with rural female children aged 4–6 having the highest exposure risk mainly due to differences in dietary structure and weight distribution among these groups. Except for rural females, the other three types (urban males, rural males, rural females) gradually decrease with age, mainly due to a decrease in grape intake per unit weight as age increases. In addition, both acute and chronic dietary risks indicate that children are more susceptible to pesticide residues posing health risks than adults, as children consume more grapes per unit weight. Therefore, we should pay more attention to the dietary risks of children.

Human health may be negatively affected by pesticide exposure, but these risks can be prevented in advance. Research has shown that pesticide residues can be correspondingly reduced during the washing process [62,63,64]. In particular, when using sodium bicarbonate for cleaning [62], the effect is more pronounced. There are also studies indicating that using atmospheric plasma discharge to generate plasma-activated water (PAW) and gaseous ozone can also reduce pesticide residues on fresh fruits [64,65,66]. In addition, methods such as peeling, bleaching and heat treatment can also effectively reduce residues [67]. The use of new technologies such as pulsed electric fields, irradiation and ultrasound can also effectively degrade pesticide residues [63,67]. Therefore, it is necessary to increase awareness among consumers that fruits and vegetables should be washed before they are eaten. In addition, strengthening the research and development of new, efficient and low-toxicity alternative pesticides is also a top method to reduce the harm of pesticides to human health. Research has shown that pyraclostrobin can reduce pesticide residues when changing pesticide formulations [68]. Developing new pesticides or changing pesticide formulations can reduce pesticide residues and minimize harm to human health. In addition, the occurrence of grape downy mildew can be controlled by replacing traditional pesticides with biopesticides in order to reduce the impact of traditional pesticides on human health. Research has shown that fungi such as *S. viridosporus*, *T. harzianum*, *Ochrobactrum* sp. and *Fusarium proliferatum* can effectively control the occurrence of downy mildew [69,70,71,72]. Some biopesticides can also improve growth, yield and fruit quality. Therefore, biopesticides are considered the best choice to replace traditional pesticides due to their advantages, such as their environmental friendliness, safety, unique chemical composition and mode of action [73,74,75]. Moreover, novel pesticides guided by molecular targets and chiral catalysts may also reduce consumer dietary risks. Finally, current pesticide risk assessments are limited in their monitoring of children and women of childbearing age; it is recommended to establish new regulatory frameworks for these special groups to fully protect public health. Dietary risk assessments in our country are mainly chronic dietary risk assessments using deterministic models as well as acute and probabilistic models. There is limited consideration of the risk of combined exposure to pesticide residues. Therefore, it may be necessary to promote new assessment methods, such as probabilistic-model-based dietary risk assessment methods to obtain more accurate assessment results. Due to the higher requirements and complexity of probabilistic models for data, it is possible to refer to the European Union to construct a graded pesticide residue risk assessment system in China that can optimize assessment results while reducing unnecessary workloads. In addition, it can also be seen from risk assessment results that divide the population that in some cases, children in China face higher dietary risks than adults. In future risk assessments, it is necessary to consider differences in population composition, such as age and geography. More comprehensive statistical data and more detailed population segmentation will help improve the accuracy of the assessment results and make the risk assessment results more in line with the actual situation. In a word, although some pesticides have high dietary risks, pesticide residues can be reduced through physical or chemical cleaning and the development of new pesticides, while strengthening the daily supervision of pesticide residues.

## 4. Conclusions

A mixed method for the determination of pyraclostrobin, cyazofamid and CCIM in grape matrices was established by sensitive and effective QuEChERS-HPLC-UV testing. This method is advantageous in terms of its simple operation, high sensitivity and short time. The final residues of pyraclostrobin and cyazofamid in grapes were <0.05–1.88 mg/kg and <0.05–0.31 mg/kg, respectively, which were lower than the maximum residue limit (MRL) in China: pyraclostrobin is 2.0 mg/kg and cyazofamid is 1.0 mg/kg. The half-lives of pyraclostrobin and cyazofamid were 17.8 d–28.9 d and 4.3 d–7.8 d. The cumulative dietary risk quotient results for pyraclostrobin and cyazofamid in grape were 0.348–5.122% and 0.249–4.334%, respectively. Girls aged 3–4 in rural areas have the highest risk, so more attention needs to be paid to them. Using probability models, the total chronic risk values for pyraclostrobin and cyazofamid were calculated to be 0.112–189.617% and 0.021–1.714%, respectively. From the results, it can be seen that the cumulative dietary exposure of pyraclostrobin and cyazofamid is significantly higher for vulnerable groups, such as children and adolescents, than for the adult population. The main reason for this is that although the dietary structure varies among different age groups based on current statistical data, it is not sufficient to compensate for the differences in toxicity tolerance caused by different body weights. Therefore, it is necessary to categorize consumer groups, especially vulnerable groups, when assessing dietary exposure risks.

## Data Availability

The data are contained within the article.

## Supplementary Materials

The following supporting information can be downloaded at: https://www.mdpi.com/article/10.3390/foods13020314/s1, Figure S1: Structures of pyraclostrobin, cyazofamid and CCIM: (A) pyraclostrobin, (B) cyazofami, (C) CCIM. Table S1: Gradient elution conditions. Table S2: Recoveries of pyraclostrobin and cyazofamid and CCIM in grapes (*n* = 5). Table S3: Probability model calculation for acute dietary risk of pyraclostrobin and cyazofamid in grape. Table S4: Chronic dietary risk assessment of pyraclostrobin in grape. Table S5: Chronic dietary risk assessment of cyazofamid in grape.
