# Supplementary material for: Dissipation, Residue and Human Dietary Risk Assessment of Pyraclostrobin and Cyazofamid in Grapes Using an HPLC-UV Detector
Source: Foods. 2024 Jan 18;13(2):314. doi: 10.3390/foods13020314 (PMC10814842; doi:10.3390/foods13020314)
Supplement: Supplementary file 1 [file foods-13-00314-s001.zip › foods-2774396-supplementary.pdf]

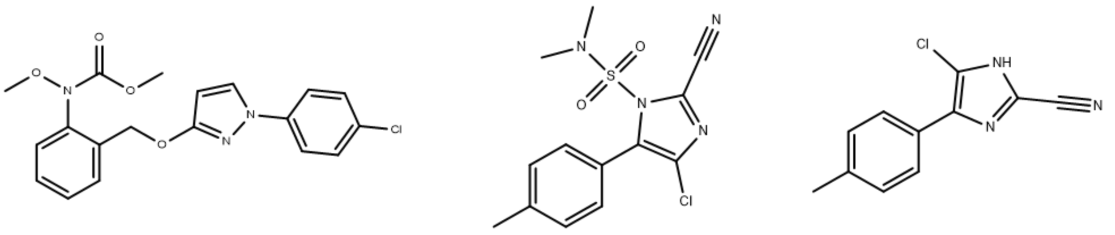

A B C

Figure S1. Structures of pyraclostrobin, cyazofamid and CCIM: (A) pyraclostrobin, (B) cyazofamid, (C) CCIM.

Table S1. Gradient elution conditions.

t/min	Flow rate (mL/min)	Acetonitrile (%)	0.1% Formic acid-water (%)
0	1	65	35
20	1	65	35

Table S2. Recoveries of pyraclostrobin and cyazofamid and CCIM in grapes ($n = 5$).

Compound	Spiked level (mg/kg)	Spike solvent average recoveries (%)	RSD (%)	Spike matrix average recoveries (%)	RSD (%)
Pyraclostrobin	0.05	93	20.3	98	19.8
	0.50	84	9.46	99	2.4
	2.0	94	6.0	99	6.9
Cyazofamid	0.05	92	10.5	97	8.1
	0.50	96	2.4	84	3.2
	1.0	98	8.2	100	1.9
CCIM	0.05	104	1.3	102	1.1
	0.50	100	4.0	99	1.3
	1.0	99	3.2	96	0.5

Table S3. Probability model calculation for acute dietary risk of pyraclostrobin and cyazofamid in grape.

pesticide	category	P50	P90	P95	P99	P99.9
pyraclostrobin	general population	8.100%	30.904%	49.706%	78.505%	139.332%
	children	17.177%	65.539%	105.415%	166.490%	295.489%
	Women of childbearing age	4.272%	16.298%	26.214%	41.402%	73.481%
cyazofamid	general population	0.852%	1.244%	1.415%	1.600%	1.872%
	children	1.808%	2.637%	3.001%	3.393%	3.970%
	Women of childbearing age	0.450%	0.656%	0.746%	0.844%	0.987%

Table S4. Chronic dietary risk assessment of pyraclostrobin in grape.

Pyraclostrobin		Deterministic model		Probabilistic model									
		urban	rural	urban					rural				
Gender	Age	mean	mean	P50	P90	P95	P99	P99.9	P50	P90	P95	P99	P99.9
male	2-3	5.122%	3.729%	1.655%	6.314%	10.156%	28.467%	189.617%	1.205%	4.596%	7.393%	20.723%	138.032%
	4-6	3.445%	3.310%	1.113%	4.247%	6.831%	19.149%	127.549%	1.070%	4.081%	6.564%	18.398%	122.549%
	7-10	2.716%	2.218%	0.878%	3.349%	5.386%	15.097%	100.561%	0.717%	2.735%	4.398%	12.329%	82.123%
	11-13	1.892%	1.572%	0.611%	2.332%	3.751%	10.515%	70.038%	0.508%	1.938%	3.117%	8.737%	58.197%
	14-17	1.734%	0.999%	0.560%	2.138%	3.438%	9.638%	64.195%	0.323%	1.232%	1.981%	5.553%	36.985%
	18-29	1.026%	0.838%	0.331%	1.264%	2.034%	5.700%	37.969%	0.271%	1.033%	1.662%	4.659%	31.031%
	30-44	0.926%	0.634%	0.299%	1.142%	1.836%	5.147%	34.287%	0.205%	0.781%	1.256%	3.522%	23.459%
	45-59	0.989%	0.530%	0.319%	1.219%	1.961%	5.496%	36.609%	0.171%	0.654%	1.051%	2.947%	19.630%
	60-69	1.149%	0.538%	0.371%	1.416%	2.278%	6.385%	42.531%	0.174%	0.663%	1.067%	2.991%	19.925%
	≥70	1.286%	0.348%	0.415%	1.585%	2.549%	7.146%	47.601%	0.112%	0.429%	0.690%	1.933%	12.877%
female	2-3	4.027%	4.484%	1.301%	4.964%	7.985%	22.382%	149.087%	1.449%	5.528%	8.891%	24.923%	166.013%
	4-6	4.170%	3.644%	1.347%	5.141%	8.268%	23.177%	154.379%	1.177%	4.492%	7.226%	20.254%	134.909%
	7-10	3.121%	2.186%	1.008%	3.848%	6.189%	17.348%	115.551%	0.706%	2.694%	4.334%	12.148%	80.917%

11-13	1.963%	1.468%	0.634%	2.420%	3.893%	10.911%	72.679%	0.474%	1.809%	2.910%	8.156%	54.328%
14-17	2.254%	1.247%	0.728%	2.778%	4.469%	12.526%	83.437%	0.403%	1.537%	2.472%	6.929%	46.154%
18-29	1.794%	1.080%	0.580%	2.211%	3.557%	9.970%	66.411%	0.349%	1.331%	2.141%	6.001%	39.973%
30-44	1.562%	0.828%	0.505%	1.925%	3.096%	8.679%	57.808%	0.268%	1.021%	1.642%	4.604%	30.665%
45-59	1.348%	0.647%	0.436%	1.662%	2.673%	7.493%	49.913%	0.209%	0.798%	1.283%	3.598%	23.964%
60-69	1.416%	0.577%	0.457%	1.745%	2.807%	7.870%	52.419%	0.187%	0.712%	1.145%	3.210%	21.379%
≥70	1.176%	0.352%	0.380%	1.449%	2.331%	6.535%	43.530%	0.114%	0.434%	0.698%	1.955%	13.023%

Table S5. Chronic dietary risk assessment of cyazofamid in grape.

Cyazofamid		Deterministic model		Probabilistic model									
		urban	rural	urban					rural				
Gender	Age	mean	mean	P50	P90	P95	P99	P99.9	P50	P90	P95	P99	P99.9
male	2-3	4.334%	3.155%	0.307%	0.553%	0.666%	0.967%	1.714%	0.224%	0.402%	0.485%	0.704%	1.248%
	4-6	2.915%	2.801%	0.207%	0.372%	0.448%	0.650%	1.153%	0.198%	0.357%	0.430%	0.625%	1.108%
	7-10	2.299%	1.877%	0.163%	0.293%	0.353%	0.513%	0.909%	0.133%	0.239%	0.288%	0.419%	0.742%
	11-13	1.601%	1.330%	0.113%	0.204%	0.246%	0.357%	0.633%	0.094%	0.170%	0.204%	0.297%	0.526%
	14-17	1.467%	0.845%	0.104%	0.187%	0.225%	0.327%	0.580%	0.060%	0.108%	0.130%	0.189%	0.334%
	18-29	0.868%	0.709%	0.061%	0.111%	0.133%	0.194%	0.343%	0.050%	0.090%	0.109%	0.158%	0.281%
	30-44	0.784%	0.536%	0.056%	0.100%	0.120%	0.175%	0.310%	0.038%	0.068%	0.082%	0.120%	0.212%
	45-59	0.837%	0.449%	0.059%	0.107%	0.129%	0.187%	0.331%	0.032%	0.057%	0.069%	0.100%	0.177%
	60-69	0.972%	0.455%	0.069%	0.124%	0.149%	0.217%	0.384%	0.032%	0.058%	0.070%	0.102%	0.180%
	≥70	1.088%	0.294%	0.077%	0.139%	0.167%	0.243%	0.430%	0.021%	0.038%	0.045%	0.066%	0.116%
female	2-3	3.408%	3.795%	0.241%	0.434%	0.524%	0.760%	1.348%	0.269%	0.484%	0.583%	0.846%	1.501%
	4-6	3.529%	3.084%	0.250%	0.450%	0.542%	0.787%	1.396%	0.218%	0.393%	0.474%	0.688%	1.220%
	7-10	2.641%	1.850%	0.187%	0.337%	0.406%	0.589%	1.045%	0.131%	0.236%	0.284%	0.412%	0.732%

11-13	1.661%	1.242%	0.118%	0.212%	0.255%	0.370%	0.657%	0.088%	0.158%	0.191%	0.277%	0.491%
14-17	1.907%	1.055%	0.135%	0.243%	0.293%	0.425%	0.754%	0.075%	0.135%	0.162%	0.235%	0.417%
18-29	1.518%	0.914%	0.108%	0.194%	0.233%	0.339%	0.600%	0.065%	0.116%	0.140%	0.204%	0.361%
30-44	1.321%	0.701%	0.094%	0.168%	0.203%	0.295%	0.523%	0.050%	0.089%	0.108%	0.156%	0.277%
45-59	1.141%	0.548%	0.081%	0.145%	0.175%	0.254%	0.451%	0.039%	0.070%	0.084%	0.122%	0.217%
60-69	1.198%	0.489%	0.085%	0.153%	0.184%	0.267%	0.474%	0.035%	0.062%	0.075%	0.109%	0.193%
≥70	0.995%	0.298%	0.070%	0.127%	0.153%	0.222%	0.394%	0.021%	0.038%	0.046%	0.066%	0.118%
